# Supplementary material for: The interactome of intact mitochondria by cross-linking mass spectrometry provides evidence for coexisting respiratory supercomplexes
Source: Mol Cell Proteomics. 2017 Dec 8;17(2):216–32. doi: 10.1074/mcp.RA117.000470 (PMC5795388; doi:10.1074/mcp.RA117.000470)
Supplement: Supplemental Data [file supp_RA117.000470_133922_0_supp_23978_fzffwf.zip › spectra_annotation/mito_spectra_annotation/186-1-1-1-5-1.pdf]

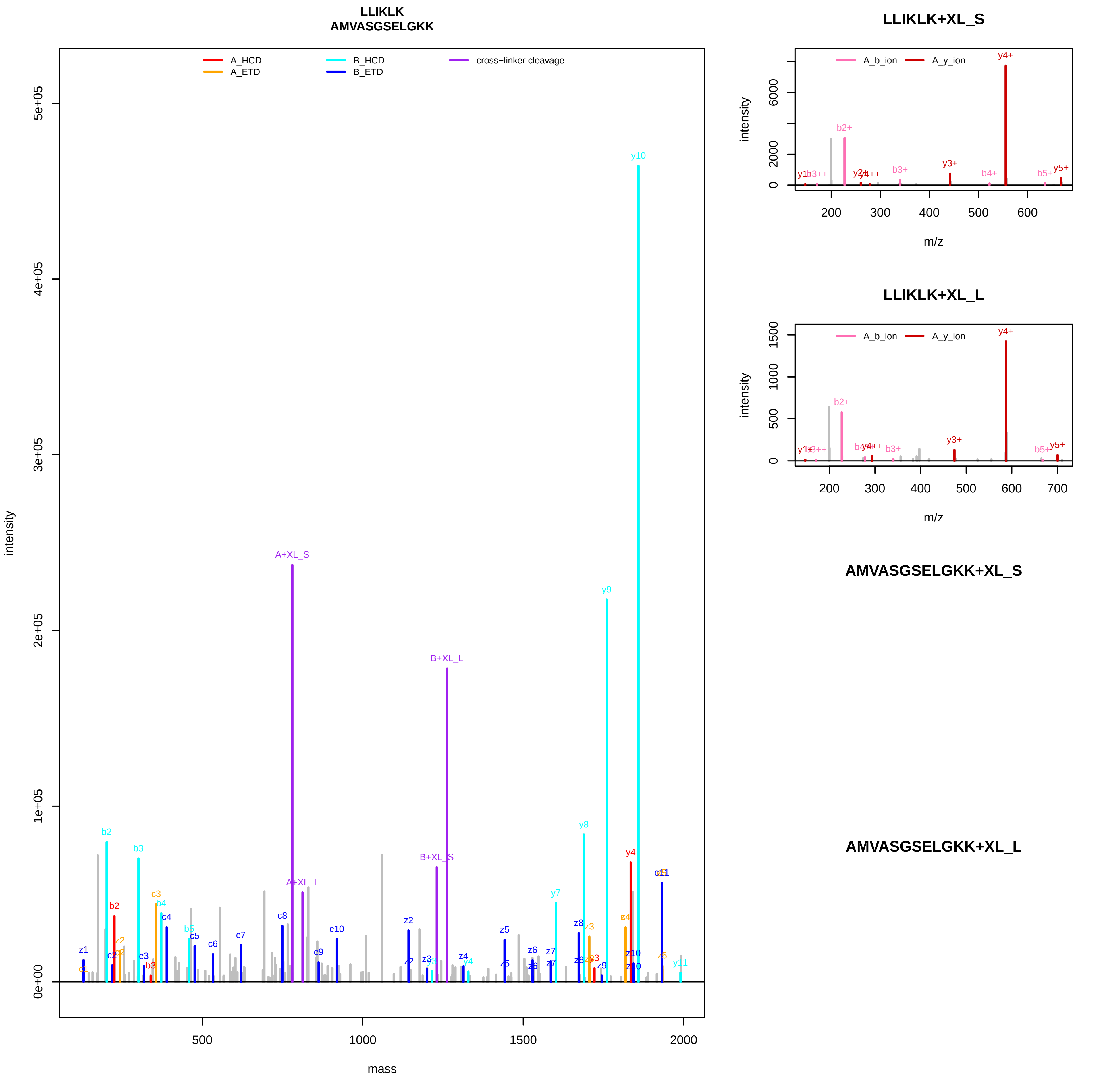
LLIKLK
AMVASGSELGKK
A_HCD
A_ETD
B_HCD
B_ETD
cross−linker cleavage
intensity
mass
A+XL_S
A+XL_L
B+XL_S
B+XL_L
y10
y9
y8
y7
y4
b2
b3
b4
c3
c4
c5
c6
c7
c8
c10
z2
z5
z8
LLIKLK+XL_S
A_b_ion
A_y_ion
y4+
b2+
y3+
b3+
b4+
b5+
y5+
m/z
intensity
LLIKLK+XL_L
AMVASGSELGKK+XL_S
AMVASGSELGKK+XL_L
